# Efficient inhibition of lung cancer in murine model by plasmid-encoding VEGF short hairpin RNA in combination with low-dose DDP

**DOI:** 10.1186/1756-9966-29-56

**Published:** 2010-05-25

**Authors:** Yong P Ma, Yang Yang, Shuang Zhang, Xiang Chen, Na Zhang, Wei Wang, Zhi X Cao, Yu Jiang, Xia Zhao, Yu Q Wei, Hong X Deng

**Affiliations:** 1 State Key Laboratory of Biotherapy, West China Hospital and West China Medical School, Sichuan University, Chengdu, Sichuan, China

## Abstract

**Background:**

VEGF is a well-validated target for antiangiogenic intervention in cancer. To date, RNAi technology has been proven to be a promising approach for targeted therapy. DDP is frequently used as a first-line drug in chemotherapy for lung cancer but usually causes severe toxicity. In this study, we investigated a novel strategy of administering and combining RNAi mediated VEGF-targeted therapy with DDP for treatment of lung cancer, with the aim of increasing efficacy and decreasing toxicity.

**Methods:**

In this study, a plasmid encoding VEGF shRNA was constructed to knockdown VEGF both in vitro and in vivo. In vitro, specificity and potency of the targeting sequence were validated in A549 lung adenocarcinoma cells by RT-PCR and ELISA assays. In vivo, therapy experiments were conducted on nude mice bearing A549 xenograft tumors. The VEGF shRNA expressing plasmids were administered systemically in combination with low-dose DDP on a frequent basis. The tumor volume and weight were measured. MVD, the number of apoptotic cells and proliferation index in tumor tissues were assessed by CD31, TUNEL and PCNA immunostaining.

**Results:**

The VEGF shRNA was highly effective in attenuating VEGF expression both in vitro and in vivo. The treatment with the VEGF shRNA alone reduced the mean tumor weight by 49.40% compared with the blank control (P < 0.05). The treatment with the VEGF shRNA plus DDP yielded maximal benefits by reducing the mean tumor weight by 83.13% compared with the blank control (P < 0.01). The enhanced antitumor efficacy was associated with decreased angiogenesis and increased induction of apoptosis.

**Conclusions:**

Our study demonstrated synergistic antitumor activity of combined VEGF shRNA expressing plasmids and low-dose DDP with no overt toxicity, suggesting potential applications of the combined approach in the treatment of lung cancer.

## Background

Lung cancer is the leading cause of cancer-related death. NSCLC accounts for 80%-85% of all lung cancers [1]. Approximately 75% of lung carcinoma patients are diagnosed with locally advanced or metastatic disease. Most of those diagnosed with early-stage disease experience relapse and the majority of them eventually die from metastatic disease [1,2]. Despite intensive efforts in treatment practices, the survival rate for lung cancer has not improved substantially in the past 25 years, resulting in a 5-year survival rate of approximately 15% [1]. Clinical outcomes have reached a plateau in survival for which new therapeutic strategies may exert benefits.

It is well known that the growth, persistence and metastasis of solid tumors are angiogenesis-dependent, so antiangiogenic therapy offers hope for treatment of solid tumors, including NSCLC [3]. Recent advances in the knowledge of tumor angiogenesis have shed light on the pivotal role of VEGF [4,5]. VEGF functions mostly as an endothelial cell-specific mitogen which mediates numerous changes within the tumor vasculature, including endothelial cell survival, proliferation, migration, vascular permeability and vasodilation [4]. Recognition of the VEGF pathway as a pivotal regulator of tumor angiogenesis has induced the development of various VEGF-targeted agents. These agents include neutralizing antibodies to VEGF or its receptors [6], tyrosine kinase inhibitors (TKIs) for VEGFRs [7], soluble antagonists for VEGF or VEGFRs [8] and so on. Some of them have been tested in the clinic. However, a large proportion of existing VEGF-targeted agents were found to have modest efficacy, when used singly in treatment of various cancers except for certain specific types of malignancy. They have thus mainly been used in combination with chemotherapy or radiotherapy. An example of this is bevacizumab (Avastin), a humanized monoclonal antibody to VEGF, which is only of benefit for patients with NSCLC when combined with conventional chemotherapy [9]. Investigations are underway with the aim of exploring more effective ways of administering and combining anti-VEGF agents with chemotherapeutic drugs.

Chemotherapy has dominated systemic therapy of cancer for a long time. In the setting of metastatic disease, chemotherapy used to be the only available approach. For NSCLC, DDP-based regimen remains the mainstay of chemotherapeutic treatment of patients with either resected or locally advanced or, metastatic diseases [2,10]. DDP-based regimens often cause severe toxic side effects, including myelosuppression, asthenia and gastrointestinal disorder, as well as long-term cardiac, renal and neurological consequences. These adverse events usually cause drug discontinuation, poor tolerance and limited therapeutic efficacy [11,12]. Preclinical and clinical studies are in progress to test various dosing/scheduling strategies for chemotherapy to increase efficacy and decrease toxicity.

Thus far, most existing VEGF-targeted agents belong to the category of recombinant protein. However, RNAi technology has been proven to be a promising alternative approach for targeted therapy and various RNAi tools are under intensive investigation. In this study, we investigated a novel strategy of administering and combining RNAi mediated VEGF-targeted therapy with DDP for treatment of lung cancer.

## Methods

### Construction of shRNA expressing plasmid

A plasmid-based shRNA expression system was used to endogenously express shRNA in human cancer cells. The targeted sequence of human VEGF: 5'-AAA CCU CAC CAA GGC CAG CAC-3' (21 nt) was selected according to a previous study [13]. The control sequence which was named HK: 5'-GAC TTC ATA AGG CGC ATG C-3' (19 nt) had no homology to any mammalian sequence. Recent evidence has revealed that U6 promoter is greatly superior to the other promoters in driving plasmid based shRNA expression and pU6shRNA is at least 100-fold more potent in gene silencing than corresponding siRNA on a numerical basis [14]. Thus, we elected U6 promoter to control the recombinant plasmids which were constructed and prepared as described elsewhere [15]. The resulting plasmids were named pshVEGF and pshHK, respectively. Oligonucleotide sequences of pshVEGF were: sense 5'-GAT CCA CCT CAC CAA GGC CAG CAC TTC AAG ACG GTG CTG GCC TTG GTG AGG TTT TTT TGA GCT CA-3'; antisense 5'-AGC TTG AGC TCA AAA AAA CCT CAC CAA GGC CAG CAC CGT CTT GAA GTG CTG GCC TTG GTG AGG TG-3'.

### Cell lines and transfection conditions

The A549 cell line was purchased from the American Type Culture Collection (ATCC, Manassas, VA, USA). The cells were cultured in RPMI1640 medium (Life Technologies, Bedford, MA, USA) supplemented with 10% fetal bovine serum and 100 U/ml penicillin and 100 U/ml streptomycin. All the Cells were maintained in a humidified atmosphere of 5% CO_2 _at 37°C.

Cell transfection was performed using FugeneHD (Roche, Mannheim, Germany) according to the manufacturer's recommendation. Briefly, A549 cells were seeded in 6-well plates at a density of 3 × 10^5 ^cells/well and cultured to reach 70-80% confluence. Two μg plasmid DNA (pshVEGF or pshHK) and 5 μl FugeneHD diluted in serum-free medium were mixed and the complex was added to the cell cultures. Growth medium was used as the control agent. The cells and the supernatants were harvested 48 h after transfection for semiquantitative RT-PCR and ELISA assays. All the transfections were performed in triplicate.

### Semiquantitative RT-PCR and ELISA assays

Total RNA was extracted from the cells with Trizol Reagent (Invitrogen, Grand Island, NY, USA). RNA concentration was measured by spectrophotometry. RT-PCR was performed with the isolated total RNA (1 μg) using TaKaRa Onestep RNA PCR Kit (Takara, Japan). β-actin was amplified as the internal control. The primers for VEGF were: forward, 5'-ATC ACG AAG TGG TGA AGT TC-3'; reverse, 5'-TGC TGT AGG AAG CTC ATC TC-3'. The expected sizes of PCR products are 265 bp for VEGF and 512 bp for β-actin [16]. VEGF and β-actin cDNA were amplified by 30 cycles of denaturation for 2 min at 94°C, annealing for 0.5 min at 62°C and extension for 0.5 min at 72°C. After the amplification, each product (10 μl) was loaded on 1% agarose gel for electrophoresis. The amplified products were quantified by Quantity One (Bio-Rad, Richmond, CA, USA). Each experiment was performed in triplicate.

Secretion of VEGF into the cell culture supernatant and tumor contents of VEGF in the A549 xenografts were determined using human VEGF ELISA Kit (Jingmei Biotech, Wuhan, China) according to the manufacturer's instructions. The results of the ELISA assay in the cell culture supernatants were expressed as pg/ml/10^5 ^cells. VEGF concentration in the tumors was corrected for total protein. Each experiment was performed in triplicate.

### Preparation of lipoplexes for in vivo therapy

The cationic liposome DOTAP and cholesterol were purchased from Avanti Polar Lipids (Alabaster, AL, USA) and Sigma (St. Louis, MO, USA), respectively. DOTAP:Chol was prepared as described elsewhere [17]. Before tail vein injection, lipoplexes were prepared as follows: 5 μg DNA and 25 μg DOTAP:Chol were diluted respectively in 50 μl 5% GS. The DNA solution was added into the liposome solution dropwisely. The mixture was incubated at room temperature for 30 min prior to injection.

Freshly prepared lipoplexes were analyzed for particle size by Malvern Zen 600 Zetasizer (Malvern, Spectris, Worcestershire, UK). The particle sizes of the lipoplexes generally ranged between 200 nm and 300 nm.

### In vivo tumor models and systemic treatment

The following studies were approved by the Institutional Animal Care and Treatment Committee of Sichuan University (Chengdu, China). To rule out the contribution of host immune response, we used a nude mouse model. Female athymic nude mice (BALB/c, 4-6 weeks of age) were housed in standard microisolator conditions free of pathogens in accordance with institutional guidelines under approved protocols. In all the experiments, 5 × 10^6 ^A549 cells suspended in 100 μl sterile PBS were injected in right flanks of the mice. When the tumors reached a mean diameter of 4-5 mm one week later, the animals were randomly assigned into groups and the treatment was initiated. There were five groups. Each group consisted of five animals. Group 1 received 5% GS. Group 2 received pshHK lipoplex. Group 3 received pshVEGF lipoplex. Group 4 received DDP. Group 5 received the combination of the regimens of group 3 and 4. The lipoplexes were administered intravenously three times per week for four weeks. DDP (2 mg/kg) was administered intraperitoneally twice per week for two weeks, starting on the next day after the administration of pshVEGF lipoplex. Our laboratory has tested various dosages of DDP and demonstrated that the dose 5 mg/kg/week is safe and effective for mice in our laboratory. To mimic 'metronomic' chemotherapy, that is, relatively frequent administrations of relatively low doses of chemotherapy, we administered DDP at 2 mg/kg twice a week. During the course of treatment, tumor size was measured by a caliper and tumor volume was calculated using the formula: V(volume) = LW^2 ^× π/6 where " L " represents the greatest length and " W " represents the perpendicular width[18]. The animals were sacrificed after twelve times of treatment. The tumors were excised and weighed. The tumor specimens were fixed in 4% formaldehyde, embedded in paraffin, and cut in 4 μm sections for immunohistochemical analysis.

### Immunohistochemistry

Immunohistochemical analysis of VEGF, CD31 and PCNA expression were performed according to the procedure described elsewhere [15]. The primary antibodies were mouse anti-human VEGF antibody, goat anti-mouse CD31 antibody and mouse anti-human PCNA antibody ( Santa Cruz Biotechnology, Santa Cruz, CA, USA).

To quantify MVD, each slide was scanned at low power magnification (× 10-100). Two 'hot spot' areas with relatively higher number of new vessels were identified which were subsequently scanned at high power magnification (× 400). Five random fields of each 'hot pot' area were analyzed. To determine proliferation index, the number of PCNA-positive cells was counted in 10 random fields (× 400).

### In situ TUNEL assay for apoptotic cells

DNA fragmentation morphology of the tumor tissues was evaluated using DeadEnd™ Fluorometric TUNEL System (Promega, Madison, Wisc., USA) according to the manufacturer's protocol. The number of green fluorescence-positive cells was counted in 10 random fields (× 400).

### Toxicity assessment

The treatment-related toxicity was mainly evaluated by weight changes of the mice. During the whole treatment course, other toxicity indexes such as ruffling of fur, behavior, feeding, cachexia and toxic death were monitored. The tissues of organs (hearts, livers, spleens, lungs and kidneys) were fixed and embedded in paraffin. The sections of 4 μm were stained with H&E and observed by two pathologists in a blinded manner.

### Statistical analysis

Data were expressed as the mean ± SD. For comparison of individual time points, differences between groups were tested by performing one-way analysis of variance (ANOVA). All P values were two sides and P < 0.05 was considered statistically significant. The Statistical Package for the Social Sciences (SPSS version 16.0, Inc., Chicago, IL. USA) was used for all statistical analysis.

## Results

### In vitro downregulation of VEGF by pshVEGF

**Figure 1:**
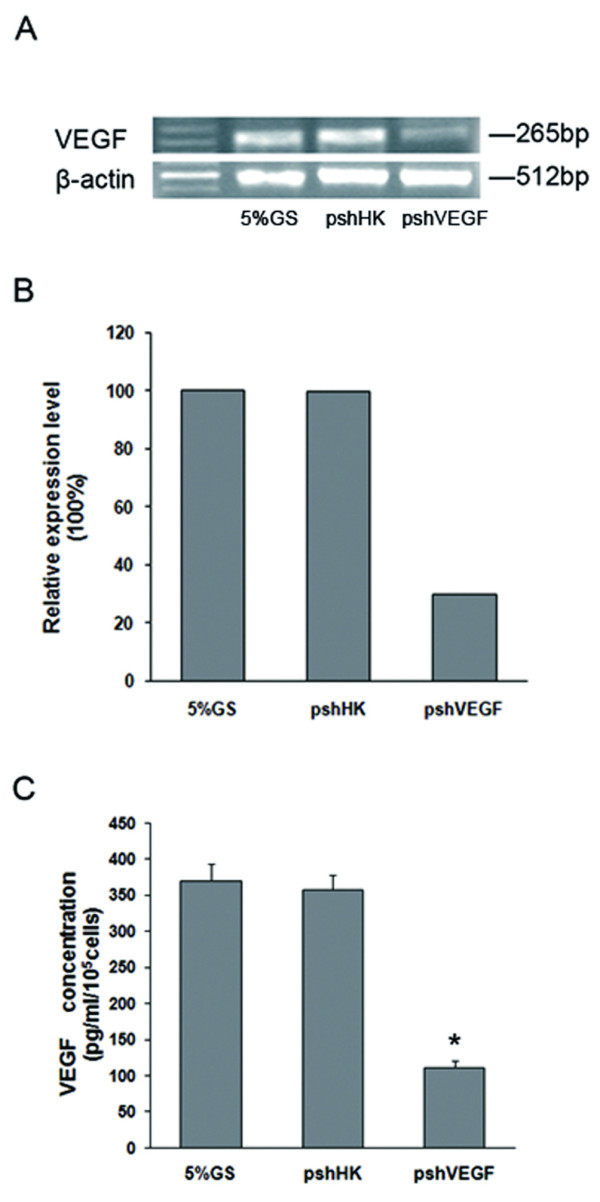
**Attenuation of VEGF expression in vitro**. **A**) Photograph of agarose gel. Cultured A549 cells were transfected with pshVEGF or pshHK. Forty-eight hours after transfection, VEGF mRNA was semiquantified by RT-PCR. The β-actin gene was used as the internal control. **B**) Densitometric analysis of VEGF mRNA was done and the relative expression of each band was normalized to the blank control (5% GS). **C**) Forty-eight hours after transfection, supernatants were collected and assayed for secreted VEGF protein by ELISA. The results are presented as mean ± SD (n = 3). P < 0.05 versus pshHK.

To evaluate specificity and potency of the targeting sequence in A549 cells, we examined its effects on VEGF expression in vitro. We first performed RT-PCR assay to measure changes in VEGF expression at the mRNA level. Cells were transiently transfected with pshVEGF and pshHK, harvested 48 h later and subjected to RT-PCR analysis. As shown in Fig. 1A.B, attenuation of VEGF expression was distinct 48 h after transfection with pshVEGF, whereas VEGF expression was not affected by pshHK. As VEGF mainly exerts its functions after it is secreted by tumor cells into the microenvironment, we then performed ELISA assay to measure the secreted VEGF protein levels in the supernatants. At 48 h posttransfection, the supernatants were collected. Cell viability, as assessed by trypan blue staining, was good (about 90%) and comparable for all experimental groups. The cells treated with liposome alone in exhibited almost the same viability as the cells in the other groups, indicating that the liposome we used has no apparent toxicity. As shown in Fig. 1C, VEGF expression in the supernatants derived from pshVEGF transfected cells was sharply decreased, whereas significantly higher levels of VEGF in the supernatants of pshHK or mock transfected cells were detected (about 370 pg/ml/10^5 ^cells). The VEGF shRNA eventually lowered the secreted amount of VEGF by 70.32% when compared with the HK shRNA (P < 0.05), which was highly consistent with the results of RT-PCR analysis. Thus, we demonstrated that the VEGF shRNA was able to knockdown VEGF expression in A549 cells with high specificity and potency.

### In vivo therapy with the combination treatment

**Figure 2:**
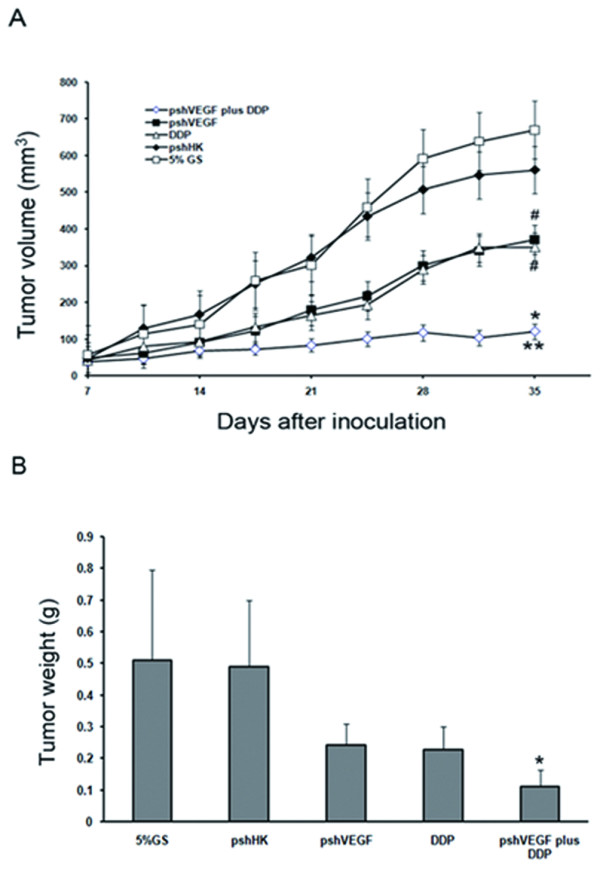
**Antitumor effect of VEGF silencing plus DDP on A549 cells in vivo**. Tumor growth curves. Each point in the curves represents the mean ± SD (n = 5 tumors). The therapy started on day 7 when the tumors reached a volume of ~50 mm^3^. The combination of VEGF silencing plus DDP enhanced the inhibition of tumor growth, #P < 0.05 versus 5% GS, *P0.05 versus pshVEGF or DDP, **P < 0.01 versus 5% GS. **B**) Weight of the tumors. Each bar represents the mean ± SD (n = 5 tumors). *P < 0.05 versus pshVEGF or DDP. Mean weights of the tumors are 0.510 g, 0.490 g, 0.242 g, 0.228 g and 0.110 g, for the 5% GS group, the pshHK group, the pshVEGF group, the DDP group and the pshVEGF plus DDP group, respectively.

**Figure 3:**
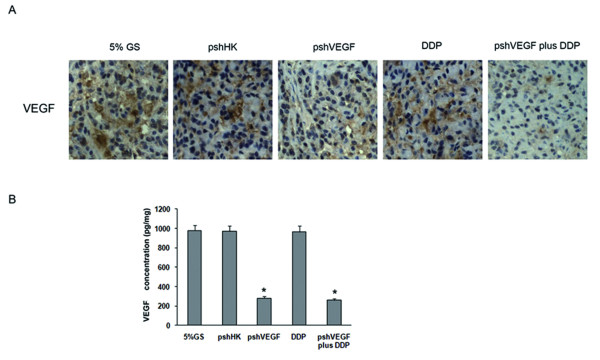
**Knockdown of VEGF expression in vivo**. **A**) Representative photographs of the tumor sections examined by immunohistochemical staining for VEGF (×400 magnification). The assessment of VEGF was based on a cytoplasmic staining pattern. **B**) The tumors were harvested and assayed for VEGF protein by ELISA. The results are presented as mean ± SD (n = 3 tumors). *P < 0.05 versus pshHK.

Having confirmed the specificity and potency of the VEGF shRNA, we then combined it with DDP in an animal model to investigate the antitumor efficacy of the combination treatment. We used a nude mice model to rule out the contribution of the host immune system. Mice bearing A549 derived tumors were treated as described in the "Methods" section. As shown in Fig. 2A.B, either pshVEGF or DDP individually effectively slowed down the tumor growth rate, reducing the tumor weight by 49.40% and 50.20% compared with 5% GS (P < 0.05). The combination treatment had a significantly enhanced antitumor effect compared with the treatment with pshVEGF or DDP alone (P < 0.05), resulting in reduction in the tumor weight by 83.13% compared with the treatment with 5% GS (P < 0.01). We didn't monitor survival because the knockdown effects may fade over time and become too modest to be examined. To prove that the therapeutic effects were related to downregulation of VEGF expression instead of other nonspecific reactions, we harvested the tumors for immunohistochemistry and ELISA assay to measure VEGF expression. As shown in Fig. 3A, we analyzed the gross distribution of immunoreactive VEGF in the tumors and observed a general decrease of VEGF staining in the tumors belonging to the mice treated with pshVEGF, whereas the tumors belonging to the mice treated with pshHK exhibited significantly more VEGF staining. Consistently, the ELISA assay showed that pshVEGF caused significant reduction in intratumoral VEGF expression compared with pshHK, as shown in Fig. 3B. Thus, we demonstrated the absence of off-target effects of the targeting sequences.

### Effect of the combination treatment on angiogenesis, cell apoptosis, and proliferation

**Figure 4:**
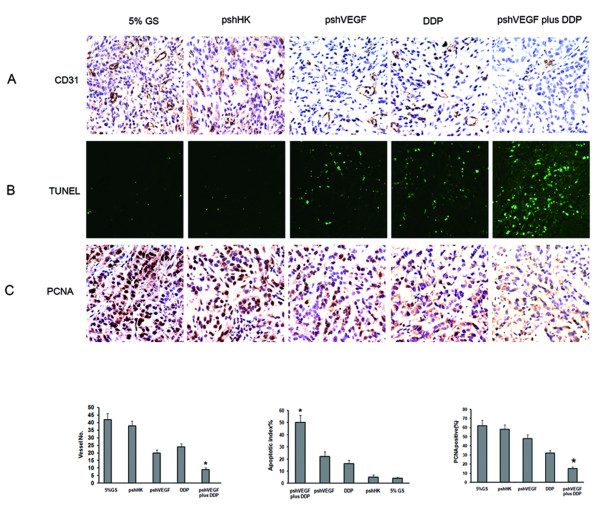
**Inhibition of tumor angiogenesis, apoptosis and proliferation by VEGF silencing plus DDP in vivo**. **A**) Representative photographs of the tumor sections examined by immunohistochemical staining for CD31 showing tumor vasculature (×400 magnification). Each bar represents the average vessel number for each group, expressed as mean ± SD. *P < 0.05 versus pshVEGF or DDP. **B**) Representative photographs of the tumor sections examined by TUNEL assay. TUNEL-positive cell nuclei (green) were observed under a fluorescence microscope (×400). Each bar represents the 'apoptosis index', expressed as mean ± SD.*P < 0.05 versus pshVEGF or DDP. **C**) Representative photographs of the tumor sections examined by immunohistochemical staining for PCNA (×400). The assessment of PCNA was based on a nuclear staining pattern. Each bar represents the ratio of PCNA positive cells to the total number of cells for each group, expressed as mean ± SD. *P < 0.05 versus pshVEGF or DDP.

To determine the mechanisms of the enhanced efficacy of the combination treatment, we examined its effects on tumor angiogenesis (MVD), tumor cell apoptosis (TUNEL) and proliferation (PCNA). We first evaluated vessel density in the harvested tumors. As shown in Fig. 4A, the mean MVD was reduced apparently in the tumors belonging to the mice treated with pshVEGF or DDP alone compared with 5% GS or pshHK. The most significant reduction in MVD occurred in the tumors of the mice receiving the combination treatment compared with pshVEGF or DDP alone (P < 0.05). Then we evaluated tumor cell apoptosis using in situ TUNEL assay. As shown in Fig. 4B, apparent cell apoptosis was identified in the tumors belonging to the mice treated with pshVEGF or DDP alone when compared with 5% GS or pshHK. The most significant apoptosis was observed in the tumors of the mice receiving the combination treatment compared with pshVEGF or DDP alone (P < 0.05). Finally, we evaluated tumor cell proliferation using PCNA staining. As shown in Fig. 4C, an apparent reduction of PCNA expression was observed in the tumors belonging to the mice treated with DDP alone compared with 5% GS or pshHK, whereas no overt reduction was observed in the tumors of the mice treated with pshVEGF alone. However, the most significant reduction of PCNA expression was observed in the tumors of the mice receiving the combination treatment compared with pshVEGF or DDP alone (P < 0.05). No significant difference in tumor angiogenesis, tumor cell apoptosis or proliferation was found between the pshHK group and the 5% GS group.

### Toxicity observation

To evaluate treatment-related toxicity, we used body weight as a surrogate for the general health status of the mice. Weight of the mice was measured regularly. The mice treated with pshVEGF, DDP and the combination of both showed a slight delay in weight gain. The mice receiving DDP or the combination treatment exhibited temporal weight loss. On the whole, there was no significant difference in body weight among the five groups. No adverse consequences in other gross measures, such as ruffled fur, strange behaviors, or toxic deaths were found in any group. Furthermore, no pathologic changes were observed in the organs (heart, liver, spleen, lung and kidney) of the mice macroscopically. Microscopic examination revealed no vascular endothelial damage, hemorrhage or edema in any organ.

## Discussion

The majority of NSCLC patients are diagnosed with late-stage disease and have poor prognosis. Clinical outcomes have reached a plateau with conventional chemotherapy as the main treatment of choice. In such clinical setting, an aggressive regimen of chemotherapy may not only fail in benefiting in survival but also harm the quality of life. To address the issue, targeted therapy was introduced. Based on advances in the knowledge of molecular events involved in NSCLC, a number of agents have been developed to specifically target signaling pathways critical to tumor progression. These rationally designed drugs were originally developed to replace conventional chemotherapy. However, numerous clinical trials have revealed the fact that a few of them managed to increase survival significantly only in combination with standard chemotherapy [19]. It appears that sole targeted therapy is not sufficient to gain benefits to the extent desired. One explanation is that when certain pathways are blocked, other pathways may compensate the loss. Another explanation is that subgroups of patients who will hopefully gain maximal benefits from targeted therapy have been far from clearly identified, therefore modest efficacy was shown in general population. A third explanation is that recombinant protein antagonists, the use of which dominates current targeted therapy, have intrinsic disadvantages that limit therapeutic efficacy [20]. At the present stage, it makes sense to design effective alternative combinatorial therapies that combine agents with novel, multiple, functionally linked properties.

The present study is a new attempt to explore a potentially effective way of administering and combining VEGF-targeted agents to first-line chemotherapeutic drugs in the treatment of NSCLC. The key findings of this study are that the combination strategy of the VEGF-targeted shRNA and low-dose DDP showed synergistic antitumor efficacy that could not be achieved with either alone, including tumor growth inhibition, neovascularization suppression and tumor apoptosis augmentation. None of serious adverse consequences, such as weight loss, strange behaviors, cachexia or toxic death, were observed.

Mechanisms of the enhanced antitumor efficacy remain to be fully elucidated, however, two mechanisms may get involved. The enhanced antitumor efficacy in vivo may be attributed to decreased angiogenesis and increased induction of apoptosis. This speculation is supported by our findings. Apparently less microvessel count and more apoptotic cells were found in the tumors belonging to the mice treated with pshVEGF plus DDP than with either alone. The first mechanism is decreased angiogenesis by the combination treatment. VEGF has been shown to function primarily via VEGFR2 which is selectively expressed on tumor endothelial cells. Several lines of evidence have revealed that binding of VEGF to VEGFR2 activates the phosphatidylinositol 3-kinase (PI3K)/AKT signaling pathway which upregulates several downstream pro-survival molecules, such as survivin, XIAP and bcl-2 [21-23]. These effectors act to shield tumor endothelial cells from various stress situations. It is known that besides tumor cells, active tumor endothelial cells are also targets of cytotoxic chemotherapeutics that were designed to kill rapidly dividing cells. Thus, deprivation of VEGF in the tumor microenvironment blocks VEGF-dependent pro-survival pathways in tumor endothelial cells and renders them more vulnerable to chemotherapeutic attacks. DDP has been found to exert its cytotoxicity to various cancer cell lines through induction of apoptosis by damaging DNA [24,25]. There is also evidence that DDP inhibits endothelial cell proliferation through suppressing DNA synthesis [26]. It appears that the proapoptotic and antiproliferating effects of DDP to endothelial cells are amplified along with the knockdown of VEGF. The knockdown of VEGF and cytotoxicity of DDP are in synergy with each other in terms of inhibiting neovascularization. The second mechanism is increased induction of apoptosis. As a result of reduced vascular density and perfusion due to inhibited angiogenesis, tumor cells are deprived of sufficient nourishments during their regrowth after chemotherapeutic insults. Meanwhile, impaired endothelium increases vascular permeability which leads to more exposure of tumor cells to chemotherapeutic drugs. The proapoptotic effects of DDP are therefore strengthened. As it is unclear whether direct effects of VEGF RNAi on the tumor cells synergized with DDP to induce apoptosis, we performed flow cytometry analysis, caspase-3 assay to detect apoptosis and MTT assay to measure cytotoxicity with the cultured cells transfected with the different plasmids (pshVEGF or pshHK), in presence and in absence of DDP. The results revealed that a) transfection with pshVEGF didn't increase cell apoptosis when compared with pshHK; b) VEGF RNAi didn't sensitize the cells to DDP in terms of inducing cell apoptosis; c) VEGF RNAi didn't significantly lower IC50 of DDP to A549 cells. These findings rule out direct synergistic effects of VEGF RNAi plus DDP on the tumor cells.

It is worth mentioning that the success in the present study is based on the dosing/scheduling strategy that was adopted for the therapy. Thus far, there are few reports describing the duration of RNAi effect on endogenous target genes [27]. On the basis of the in vitro result that attenuation of VEGF expression was distinct 48 h after plasmid transfection, the plasmids were given to the mice every other day at a dose of 5 μg. It was supposed that specific knockdown effects could be maintained and strengthened in this way without severe toxicities that have been reported to come with the use of short bursts of high-dose DNA/liposome complex [28]. Based on the same consideration about toxicity, DDP was administered in a similar way. It was given to the mice at the dose of 2 mg/kg twice a week instead of at maximum tolerated dose(9 mg/kg/week)[29]. In this study, the enhanced efficacy without overt toxicity suggested the effectiveness of the dosing/scheduling strategy.

The success of gene therapy is highly dependent on delivery vector. In this study, we elected the cationic liposome DOTAP:Chol as the delivery vector. It is a well-characterized nonviral vector and has been advanced into phase I clinical trial for treatment of NSCLC [30-32]. In this study, attenuation of VEGF expression in vivo confirmed the successful delivery of DOTAP:Chol.

## Conclusions

In summary, our study shows that the combination of plasmid-encoding VEGF shRNA and low-dose DDP is highly effective in inhibiting NSCLC growth in vivo without overt toxicity. The enhanced antitumor efficacy may be attributed to synergistic mechanisms of decreased angiogenesis and increased induction of apoptosis. Our findings suggest the potential use of the combined approach in treatment of lung cancer.

## List of abbreviations

VEGF: vascular endothelial growth factor; RNAi: RNA interference; DDP: cisplatin; shRNA: short hairpin RNA; RT-PCR: reverse transcription polymerase chain reaction; ELISA: enzyme-linked immunosorbent assay; MVD: microvessel density; shRNA: short hairpin RNA; TUNEL: terminal deoxynucleotidyl transferase dUTP nick end labeling; PCNA: proliferating cell nuclear antigen; NSCLC: non-small cell lung cancer; VEGFR: vascular endothelial growth factor receptor; siRNA: small interfering RNA; DOTAP: 1, 2 dioleoyl-3-trimethylammonium-propane; GS: glucose solution; PBS: phosphate-buffered saline; SD: standard deviation; MTT: method of transcriptional and translational.

## Competing interests

The authors declare that they have no competing interests.

## Authors' contributions

YPM and YY designed the procedure of the study, carried out the plan and drafted the manuscript. HXD, SZ and ZXC participated in cell culture, animal experiments and immunohistological analysis. XC assisted to synthetise and formulate the lipoplexes. NZ, WW helped to construct and prepare the therapeutic plasmids. YJ and XZ assisted in immunohistological analysis. YQW supervised the whole experimental work and revised the manuscript. All authors read and approved the manuscript.
